# Impact of diuretic therapy-associated electrolyte disorders present on admission to the emergency department: a cross-sectional analysis

**DOI:** 10.1186/1741-7015-11-83

**Published:** 2013-03-27

**Authors:** Spyridon Arampatzis, Georg-Christian Funk, Alexander Benedikt Leichtle, Georg-Martin Fiedler, Christoph Schwarz, Heinz Zimmermann, Aristomenis Konstantinos Exadaktylos, Gregor Lindner

**Affiliations:** 1 Department of Emergency Medicine, Inselspital, University Hospital Bern, Freiburgstrasse, Bern, 3010, Switzerland; 2 Department of Nephrology and Hypertension, Inselspital, University Hospital Bern, Bern, Switzerland; 3 Department of Respiratory and Critical Care Medicine, Otto Wagner Hospital and Ludwig Boltzmann Institute for COPD and Respiratory Epidemiology, Vienna, Austria; 4 Center of Laboratory Medicine, Inselspital, University Hospital Bern, Bern, Switzerland; 5 Department of Nephrology, Medical University of Graz, Graz, Austria

**Keywords:** Diuretics, Electrolyte disorders, Outcome

## Abstract

**Background:**

Diuretics are among the most commonly prescribed medications and, due to their mechanisms of action, electrolyte disorders are common side effects of their use. In the present work we investigated the associations between diuretics being taken and the prevalence of electrolyte disorders on admission as well as the impact of electrolyte disorders on patient outcome.

**Methods:**

In this cross sectional analysis, all patients presenting between 1 January 2010 and 31 December 2011 to the emergency room (ER) of the Inselspital, University Hospital Bern, Switzerland were included. Data on diuretic medication, baseline characteristics and laboratory data including electrolytes and renal function parameters were obtained from all patients. A multivariable logistic regression model was performed to assess the impact of factors on electrolyte disorders and patient outcome.

**Results:**

A total of 8.5% of patients presenting to the ER used one diuretic, 2.5% two, and 0.4% three or four. In all, 4% had hyponatremia on admission and 12% hypernatremia. Hypokalemia was present in 11% and hyperkalemia in 4%. All forms of dysnatremia and dyskalemia were more common in patients taking diuretics. Loop diuretics were an independent risk factor for hypernatremia and hypokalemia, while thiazide diuretics were associated with the presence of hyponatremia and hypokalemia. In the Cox regression model, all forms of dysnatremia and dyskalemia were independent risk factors for in hospital mortality.

**Conclusions:**

Existing diuretic treatment on admission to the ER was associated with an increased prevalence of electrolyte disorders. Diuretic therapy itself and disorders of serum sodium and potassium were risk factors for an adverse outcome.

## Background

Diuretics are a mainstay of therapy for a wide variety of diseases ranging from hypertension to the nephrotic syndrome. Despite this key role, the various substances used as diuretics can have serious side effects, such as volume depletion, ototoxicity as with loop diuretics, and, of course, the induction of electrolyte disorders [1]. In an ageing population, the number of patients treated with diuretics for different indications is increasing, as is the relevance of their side effects [2,3].

Studies investigating diuretic therapy-induced electrolyte disorders have mainly focused on the effects of thiazide diuretics. In a randomized controlled trial in hypertensive patients, serum potassium levels were significantly lower in patients treated with thiazide diuretics [4]. However, only patients who did not receive potassium supplements developed marked hypokalemia with potassium levels below 3.0 mmol/L [4]. A more recent study on hypertensive patients found an incidence of 30% for hyponatremia in patients treated with thiazide diuretics [5]. The same study did not find an increased risk of hospitalization or death in patients with thiazide-induced hyponatremia [5].

To the best of our knowledge, the prevalence of diuretic use and associated electrolyte disorders have never been investigated in a large group of patients presenting to a hospital emergency room (ER). Findings from large patient series on the impact of non-thiazide diuretics on electrolyte levels are especially lacking.

Therefore, we investigated the associations between diuretics being taken and the prevalence of electrolyte disorders as well as the impact of electrolyte disorders on patient outcome.

## Methods

All patients admitted to the ER of the Inselspital, University Hospital Bern, in whom serum sodium was measured between 1 January 2009 and 31 December 2010 were included in this cross-sectional analysis. During the study period, 28,523 serum sodium measurements were made in 22,239 patients.

For these 22,239 patients, we gathered data on age, sex, admission type (medical or surgical), country of residence, hospital admission, length of hospital stay, outcome and final diagnosis as classified by the *International Classification of Diseases*, 10th revision (ICD-10). We also screened the patients’ medications for diuretic use. We gathered information on diuretic use in accordance with the diuretic substances licensed by the Swiss Agency for Therapeutic Products (Swissmedic) as registered in the list of pharmaceutical products (http://www.kompendium.ch). Diuretic agents combined with other substance classes such as antihypertensive agents were also considered in the analysis. We acquired information on the daily dose of the following substances: hydrochlorothiazide, chlorthalidone, butizide, amiloride, spironolactone, eplerenone, furosemide, torasemide, indapamide, metolazone and acetazolamide. Also, if available, the levels of the following analytes were obtained in the patients with serum sodium measurements: potassium, chloride, calcium, phosphate, magnesium, creatinine and urea. Using the values of these variables, the patients were classed as having an electrolyte disorder or not, based on the reference ranges of our central laboratory (sodium: 135 to 145 mmol/L, potassium: 3.5 to 4.7 mmol/L, chloride: 97 to 108 mmol/L, calcium: 2.1 to 2.55 mmol/L, phosphate: 0.84 to 1.45 mmol/L, magnesium: 0.75 to 1.00 mmol/L). We used baseline characteristics and serum creatinine to calculate the estimated glomerular filtration rate in accordance with the Modified Diet in Renal Disease (MDRD) formula.

In the case of multiple admissions, only the first admission to the ER was considered for the analysis.

As this study was a retrospective analysis, there was no need for informed consent. The study was approved by the Ethics Committee of the Canton of Bern, Switzerland.

### Statistical analysis

Data are presented as means ± standard deviation (SD), medians or proportions, as appropriate. Between-group comparisons of continuous variables were performed using the Mann–Whitney U test. Proportions were compared using the χ^2^ test. When the expected count for cells was lower than five, Fisher’s exact test or the exact test module in SPSS was used.

Multivariable logistic regression analysis was used to explore the association of the various predictors with the presence of electrolyte disorders and with hospitalization. Predefined covariates were added to the logistic regression models.

The cumulative hazard of death was calculated by the Kaplan-Meier method and the log-rank test was used for comparison of groups. Cox regression was used to test associations of the electrolyte disorders with the survival time adjusted for predefined covariates. A two-sided *P* value of <0.05 was considered statistically significant for all analyses. The statistical analysis was performed using SPSS (SPSS for Windows V.17.0, Chicago, IL, USA).

## Results

A total of 22,239 patients with serum sodium measurements were included in the study. The mean age at presentation was 52 years (SD 20 years) and 57% were men. In all, 76% of patients were Swiss residents. Mean baseline laboratory values are given in Table 1.

**Table 1:** Baseline laboratory values

**Substance**	**Mean**	**Standard deviation**	**Number of patients (%)**
Creatinine	82.0	64.0	17,813 (80)
Urea	6.4	5.1	15,513 (70)
Osmolality	304.0	23.0	3,524 (16)
Sodium	139.0	4.0	22,239 (100)
Potassium	3.5	0.6	22,165 (99)
Chloride	103.0	6.0	2,170 (10)
Phosphate	0.6	0.6	2,390 (11)
Calcium	2.0	0.2	8,267 (37)
Magnesium	0.8	0.1	5,339 (24)

All values are mmol/L, except creatinine (μmol/L) and osmolality (mosm/kg).

In all, 19,725 patients (88.7%) had no diuretic agent on admission, while 1,884 (8.5%) had 1, 547 (2.5%) had 2, and 83 (0.4%) had 3 or 4 different diuretic substances as medication (Table 2). Loop diuretics were the most common, with 1,196 patients (48%) on torasemide and 231 (9%) on furosemide.

**Table 2:** Overview of diuretic substances and dosage

**Diuretic**	**Number of patients (%)**	**Median dose (mg)**	**Quartile 1**	**Quartile 3**
Torasemide	1.196 (48)	10	5	20
Furosemide	231 (9)	40	40	80
Hydrochlorothiazide	975 (39)	12.5	12.5	12.5
Chlorthalidone	102 (4)	12.5	12.5	25
Butizide	16 (1)	2.5	2.5	5
Amiloride	97 (4)	5	2.5	5
Spironolactone	403 (16)	25	25	50
Eplerenone	15 (1)	25	25	50
Indapamide	58 (2)	1.5	1.5	1.5
Metolazone	121 (5)	5	2.5	5
Acetazolamide	20 (1)	250	250	500

The mean serum sodium and serum chloride concentration were significantly lower in patients on diuretic medication on admission to the ER (138 ± 5 vs 139 ± 4 mmol/L, and 101 ± 8 vs 103 ± 6 mmol/L, *P* <0.0001). The mean serum potassium level was higher in patients on diuretics (4.03 ± 0.63 vs 3.93 ± 0.45 mmol/L, *P* <0.0001). Patients on diuretics on admission also had a significantly higher mean serum creatinine concentration (116 ± 97 vs 78 ± 56 μmol/L, *P* <0.0001). Mean MDRD was higher in the group without diuretic medication (58 ± 7 vs 51 ± 14).

In all, 845 patients (4% of patients with sodium measurements) had hyponatremia on admission, 2,630 (12%) hypernatremia, 246 (11%) hypochloremia, and 245 (11%) had hyperchloremia. Hypokalemia was present in 2,459 (11%) and hyperkalemia was found in 974 (4%). Hypophosphatemia was present in 611 (26%) patients, hyperphosphatemia in 215 (9%), hypomagnesemia in 1,308 (24%), and hypermagnesemia in 244 (5%) patients. Hypocalcemia was found in 956 (12%) and hypercalcemia in 108 (1%).

Hyponatremia was more common in patients taking diuretic medication (20% vs 7.7%, *P* <0.0001). The absolute number of different diuretics taken by patients was associated with a higher prevalence of hyponatremia (*P* <0.0001). A total of 14% of patients with hyponatremia were taking loop diuretics, 12% thiazide-type diuretics, 6% aldosterone antagonists, and 1% potassium-sparing diuretics. Hyponatremia was more likely to be seen in patients taking loop diuretics (OR 1.23), thiazide diuretics (OR 1.48), potassium-sparing diuretics (OR 1.64) and aldosterone antagonists (OR 2.45) than in patients without diuretics (*P* <0.0001). In the multivariable regression model, use of thiazide diuretics (odds ratio (OR) 1.44, *P* <0.0001) and aldosterone antagonists (OR 2.4, *P* <0.0001) were associated with the presence of hyponatremia after correction for age, sex and estimated glomerular filtration rate (eGFR) as calculated by MDRD.

Hypernatremia was more common in patients taking diuretic medication (2.2% vs 1.6%, *P* = 0.03). As with hyponatremia, the prevalence of hypernatremia rose with the number of diuretic drugs taken (*P* = 0.02). Loop diuretics were associated with a significantly higher prevalence of hypernatremia than no diuretic treatment (*P* = 0.0023). There was no significant difference for all other types of diuretic agent (*P* >0.05). Use of loop diuretics was an independent risk factor for the presence of hypernatremia after correction for age, sex and eGFR as calculated by MDRD (OR 1.68, *P* = 0.0232).

Hypokalemia was significantly more common in patients under diuretic therapy (17% vs 11%, *P* <0.0001). In patients taking loop diuretics (*P* = 0.0337), thiazide diuretics (*P* <0.0001) and potassium-sparing diuretics (*P* <0.0001), hypokalemia was more common than in patients on no diuretic therapy. No difference was seen for aldosterone antagonists (*P* = 0.7) or carboanhydrase inhibitors (*P* = 0.9). In the multivariable regression model, loop diuretics (OR 1.27, *P* = 0.0316), thiazide diuretics (OR 2.18, *P* <0.0001) and potassium-sparing diuretics (OR 2.13, *P* = 0.0038) were associated with the presence of hypokalemia. Male sex (OR 0.61, *P* <0.0001) was also associated with a lower risk for hypokalemia.

Hyperkalemia was significantly more common in patients on diuretic therapy (13% vs 4%, *P* <0.0001). The prevalence of hyperkalemia was linked to the number of diuretic agents taken by patients (*P* <0.0001). All types of diuretics were associated with an increased prevalence of hyperkalemia (*P* <0.05). In the multivariable regression model, potassium-sparing diuretics (OR 3.3, *P* = 0.0044), aldosterone antagonists (OR 1.76, *P* <0.0015) and age (OR 1.03, *P* <0.0001), male sex (OR 1.35, *P* <0.0001) and serum creatinine (OR 2.23, *P* <0.0001) were associated with the presence of hyperkalemia (a higher MDRD was protective for the presence of hyperkalemia, OR 0.93, *P* <0.0001). Thiazide diuretics were associated with a lower risk of hyperkalemia (OR 0.65, *P* = 0.0054).

In the multivariable regression model, the presence of hyponatremia (OR 1.29, *P* = 0.0117) and advanced age (OR 1.01, *P* <0.0001) were associated with a need for hospitalization, while male sex was associated with a lower risk for a need for hospitalization (OR 0.86, *P* = 0.0186).

**Figure 1:**
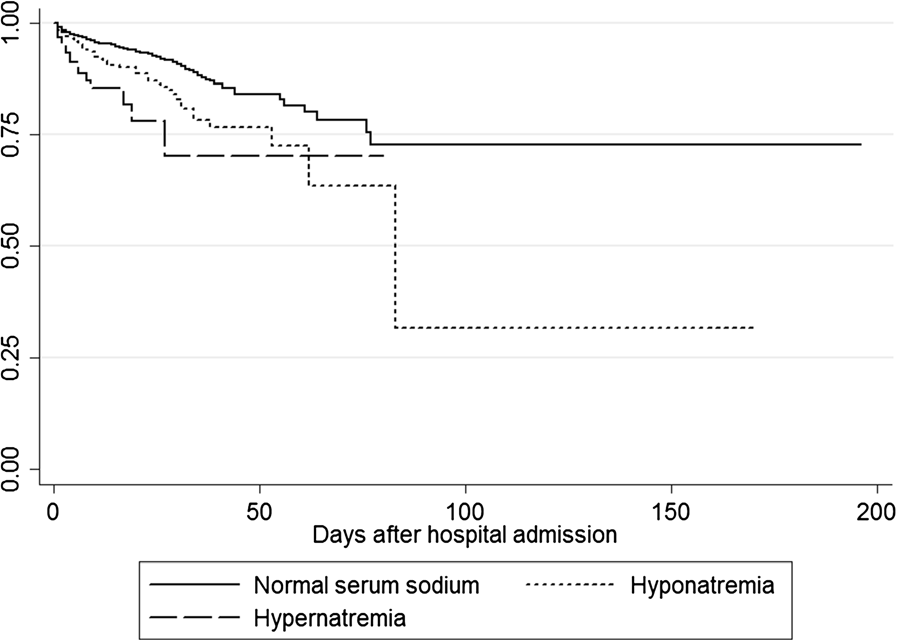
**Kaplan**-**Meier curve for mortality in patients with hyponatremia (OR 1.55, *P *=** **0.004) or hypernatremia (OR 3.21, *P *=** **0.0001) versus patients with normal serum sodium.**

**Figure 2:**
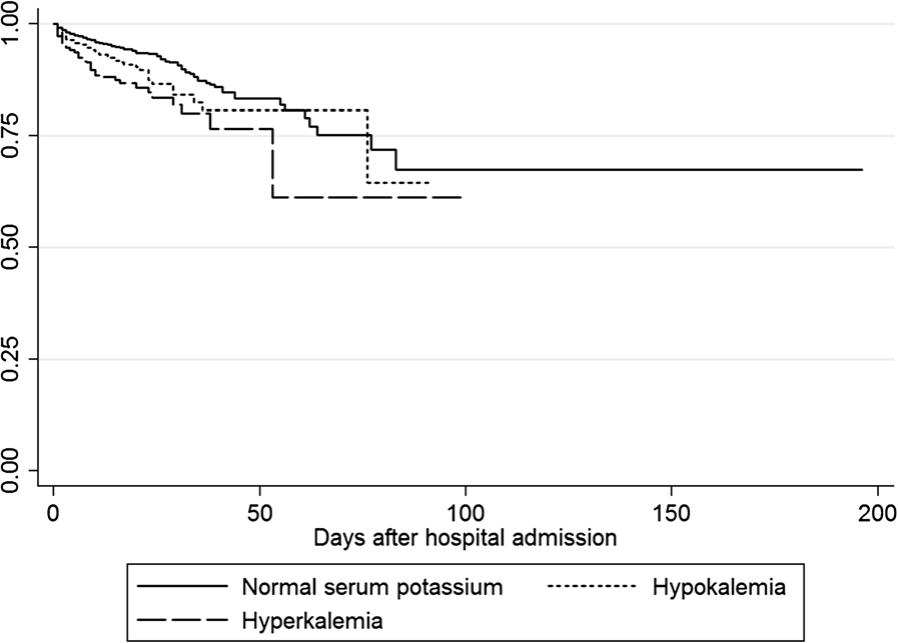
**Kaplan**-**Meier curve for mortality in patients with hypokalemia (OR 1.89, *P *<0.0001) or hyperkalemia (OR 2**.**35, *P *<0.0001) versus patients with normal serum sodium.**

Overall, in-hospital mortality was 2.6%. The in-hospital mortality of patients with hyponatremia was 6.8% and was 2.6% for patients with hypernatremia. Patients with hypokalemia had an in-hospital mortality of 4.5%, while 10.4% of patients with hyperkalemia died. Both hyponatremia (OR 1.55, *P* = 0.0003) and hypernatremia (OR 3.21, *P* <0.0001) were predictors for increased mortality in the multivariable regression model after correction for age, sex and eGFR as calculated by MDRD. The presence of hypokalemia (OR 1.89, *P* <0.0001) or hyperkalemia (OR 2.35, *P* <0.0001) on admission was also associated with higher mortality in hospital. Figures 1 and 2 show Kaplan-Meier curves for mortality in patients with dysnatremias and dyskalemias and for those with normal serum sodium concentrations.

Diuretic use and the number of diuretics on admission as well as serum creatinine and age were also associated with increased in-hospital mortality (*P* <0.0001).

## Discussion

Over a 2-year period, more than 20,000 patients seen in our ER at a large tertiary care hospital for different reasons had serum sodium measurements, which was the prime variable for inclusion in our study. A total of 11% of these patients were taking diuretic agents, and 3% were taking more than one diuretic agent.

Serum sodium levels were significantly lower in patients under diuretic treatment and significantly more patients with hyponatremia were taking a diuretic on admission. Interestingly, potassium levels were significantly higher in patients under diuretic therapy than in those without diuretics. Hypokalemia, however, was significantly more often seen in patients on diuretic agents. Loop diuretics were an independent risk factor for hypernatremia and hypokalemia and thiazide use was associated with the presence of hyponatremia and hypokalemia while they were protective against hyperkalemia. The use of aldosterone antagonists was an independent risk factor for the presence of hyponatremia and hyperkalemia. In a Cox regression model, all forms of dysnatremia and dyskalemia were independent risk factors for in-hospital mortality.

To the best of our knowledge, this is the first study investigating the associations between all types of diuretic and electrolyte levels in a large group of patients presenting to the ER of a major teaching hospital. Our finding of an association between thiazide diuretic and hyponatremia and hypokalemia confirms the results of previous studies [2,4-7]. The association between hypernatremia and loop diuretic use is explained by the mechanism of action of loop diuretics where the excretion of electrolyte-free water exceeds the increased natriuresis [8]. The mechanism of action of aldosterone antagonists explains their association with hyponatremia and hyperkalemia in the present study [8].

We found that hyponatremia and hypernatremia on admission are associated with increased mortality and this supports results from previous studies by our group [9-11]. A recent study in patients with myocardial infarction also found increased mortality in patients with hypokalemia and hyperkalemia, supporting our findings on the untoward effects of dyskalemias on outcome [12].

Although our study cannot demonstrate causality, the effects of electrolyte disorders on different physiological functions suggest an independent effect on mortality [13-17]. This puts the relevance of diuretic therapy-induced electrolyte disorders into a new light: should patients at risk of developing an electrolyte disorder from a certain diuretic be treated with a different substance? This would be an option, since risk profiles exist at least for thiazide-induced hyponatremia [18,19]. A further option would be to more strongly encourage electrolyte supplementation since this appears to be beneficial [4]. What at least seems crucial is to create awareness in physicians prescribing diuretic therapy that regular electrolyte monitoring should be performed to identify those who may develop the adverse effects of electrolyte disorders, such as an increased risk of falls or fractures [14,20].

Vasopressin antagonists are the currently newest type of diuretic medication on the market. Today, indications for vasopressin antagonists include euvolemic and hypervolemic hyponatremia [21]. With these new diuretic agents new side effects can be expected: Due to their mechanism of action, namely an inhibition of vasopressin action, overshooting “corrections” of serum sodium could occur. Additionally, hypovolemia and its effects such as dizziness were described in studies on vasopressin antagonists [22].

One of the limitations of our study is that it was not practicable to obtain data on and evaluate all medications the patients were taking because of the large number of patients included. Information on other substances that may influence serum electrolytes, such as angiotensin-converting enzyme inhibitors, was not available. We also only had information on the patient’s principal diagnosis, thus excluding secondary diagnoses as potential contributory factors, such as cirrhosis of the liver. One of the strengths of the study, however, was the large number of patients included, and we therefore do not expect that these limitations had a substantial effect on our findings.

## Conclusions

The spectrum of electrolyte disorders in patients on diuretics admitted to our ER varied with the class of diuretic. All diuretics were associated with an increased prevalence of electrolyte disorders on admission. Diuretic therapy itself and disorders of serum sodium and potassium were risk factors for an adverse outcome.

## Abbreviations

ER: Emergency room; (e)GFR: (estimated) Glomerular filtration rate; ICD-10: *International Classification of Diseases*, 10th revision; MDRD: Modified diet in renal disease.

## Competing interests

The authors declare that they have no competing interests.

## Authors’ contributions

GL had the idea for and designed the study and was involved in data gathering and analysis and performed the manuscript draft. G-CF performed the statistical analysis and helped with the manuscript draft and with the revisions. SA was involved in the conception of the study and data analysis and helped with the draft of the manuscript. AB gathered data and performed parts of the statistical analysis. G-MF participated in data gathering, statistical analysis and the manuscript draft. CS performed data analysis and data quality assurance and helped with the manuscript draft. HZ participated in the manuscript draft and data quality assurance. AKE was involved in the conception of the study and the manuscript draft. All authors gave their final approval to the manuscript.

## Pre-publication history

The pre-publication history for this paper can be accessed here:

http://www.biomedcentral.com/1741-7015/11/83/prepub
